# Bridging archaeology and marine conservation in the Neotropics

**DOI:** 10.1371/journal.pone.0285951

**Published:** 2023-05-25

**Authors:** Thiago Fossile, Dannieli Firme Herbst, Krista McGrath, Alice Toso, Paulo César Fonseca Giannini, Rafael Guedes Milheira, Simon-Pierre Gilson, Jessica Ferreira, Dione da Rocha Bandeira, Manuel Haimovici, Bruna Ceretta, Mariana G. Bender, André Carlo Colonese

**Affiliations:** 1 Department of Prehistory and Institute of Environmental Science and Technology (ICTA-UAB), Universitat Autònoma de Barcelona, Cerdanyola del Vallès, Barcelona, Spain; 2 BoCAS, Bonn Center for ArchaeoSciences, Institut für Archäologie und Kulturanthropologie, Rheinische Friedrich-Wilhelms-Universität, Bonn, Germany; 3 Instituto de Geociências, Universidade de São Paulo, São Paulo, Brazil; 4 Departamento de Antropologia e Arqueologia, Universidade Federal de Pelotas, Pelotas, Brazil; 5 Instituto de Ciências Humanas e da Informação, Universidade Federal do Rio Grande, Rio Grande, Brazil; 6 Programa em Patrimônio Cultural e Sociedade, Universidade da Região de Joinville, Joinville; Museu Arqueológico de Sambaqui de Joinville, Joinville, Brazil; 7 Universidade Federal do Rio Grande, Instituto de Oceanografia, Rio Grande, RS, Brazil; 8 Laboratório de Macroecologia e Conservação Marinha, Universidade Federal de Santa Maria, Santa Maria, Rio Grande do Sul, Brazil; MARE – Marine and Environmental Sciences Centre, PORTUGAL

## Abstract

Anthropogenic impacts on tropical and subtropical coastal environments are increasing at an alarming rate, compromising ecosystem functions, structures and services. Understanding the scale of marine population decline and diversity loss requires a long-term perspective that incorporates information from a range of sources. The Southern Atlantic Ocean represents a major gap in our understanding of pre-industrial marine species composition. Here we begin to fill this gap by performing an extensive review of the published data on Middle and Late Holocene marine fish remains along the southern coast of Brazil. This region preserves archaeological sites that are unique archives of past socio-ecological systems and pre-European biological diversity. We assessed snapshots of species compositions and relative abundances spanning the last 9500 years, and modelled differences in species’ functional traits between archaeological and modern fisheries. We found evidence for both generalist and specialist fishing practices in pre-European times, with large body size and body mass caught regularly over hundreds of years. Comparison with modern catches revealed a significant decline in these functional traits, possibly associated with overfishing and escalating human impacts in recent times.

## Introduction

The rapid decline of global biodiversity is one of the most severe and escalating issues of our time [1, 2], increasing at an alarming rate in coastal and ocean ecosystems through overexploitation, habitat degradation, and pollution, among other stressors [3, 4]. Because taxonomic diversity (richness and abundance of species) and ecosystem function and services (the capacity of natural processes and components to provide goods and services that satisfy human needs, directly or indirectly, [5]) are positively correlated [6], biodiversity loss (e.g. taxonomic and functional diversity) as well as changes in species distribution, composition, and abundance, have potentially dramatic consequences, altering ecosystem functions and compromising food provisions and the livelihoods of people around the world [2, 7, 8]. Scholars have sought to measure the scale of Anthropocene defaunation through modern observations for decades [9, 10]; questions remain, however, about conservation and restoration targets as establishing reference baselines is complex in marine ecosystems impacted by long-term human activities [11–13], particularly in regions with conspicuous biological knowledge shortfalls, such as Brazil [14].

Brazil is a megadiverse country [15], with the majority of its population and economic activities concentrated along its ∼7,500 km coastline. In 2015, coastal and marine economies contributed to nearly 20% of the country’s annual GDP [16]. In the south, the coastal strip of the Atlantic Forest and Pampa biomes support large marine biodiversity [17] and numerous ecosystem services for human populations [18]. In particular, the Atlantic Forest is a global biodiversity hotspot [19, 20] and a priority region for efforts of ecosystem restoration and biodiversity adaptation to climate change [1, 21]. However, in the last decades, population growth, increasing urbanisation, industrialisation, tourism and agricultural expansion have caused significant impacts on coastal environments in these regions [22–24]. The southern region, comprising the states of Paraná, Santa Catarina, and Rio Grande do Sul, has historically been the largest territory of fish exploitation in Brazil [25], and thus is a strategic area for marine conservation within the context of a sustainable blue economy and blue growth. Yet several economically important demersal fish species are currently threatened by overfishing, bycatch and habitat degradation [26, 27]. Recent studies revealed that some of these stressors have been in action for over a century [28], potentially distorting perceptions about the degree to which local organisms and environments have been altered over time [29, 30]. As a consequence, a thorough understanding of the scale of marine biodiversity loss and population decline requires knowledge of species composition, distribution and relative abundance predating the anthropogenic impacts of the past centuries [31, 32].

Although typically limited to decadal and centennial timescales, archaeological sites retain information on past biological diversity that is becoming central in debates about long-term anthropogenic impacts on ecosystems [33, 34]. South America, however, has received only cursory attention. In this region, archaeological faunal remains are some of the few sources of information on pre-European vertebrate and invertebrate diversity and relative abundance, from single species to several taxonomic and functional groups [35]. Moreover, because Indigenous environmental stewardship is considered an example of sustainable resource use [36] and key to biological conservation in tropical and subtropical regions of South America [37, 38], studies of archaeological faunal remains also offer a window into the origin and changing nature of these longstanding practices.

Indigenous groups have exploited coastal environments in the Atlantic Forest and Pampa biomes of southern Brazil since at least the Middle Holocene [39, 40], leaving behind thousands of archaeological sites containing large amounts of fish remains [41]. Archaeological fish remains are largely the product of economic strategies and related cultural practices, and thus provide the most direct evidence of which species have been selectively (e.g. due to food preferences, taboos, technology) exposed to fishing pressures in the past and over long timescales. Systematic zooarchaeological studies in these regions began in the 1970s and it is now possible to perform regional syntheses on published records for dozens of sites, obtaining snapshots of fish landings over *ca*. 9500 years of pre-European occupation, prior to the 16th century AD.

**Fig 1:**
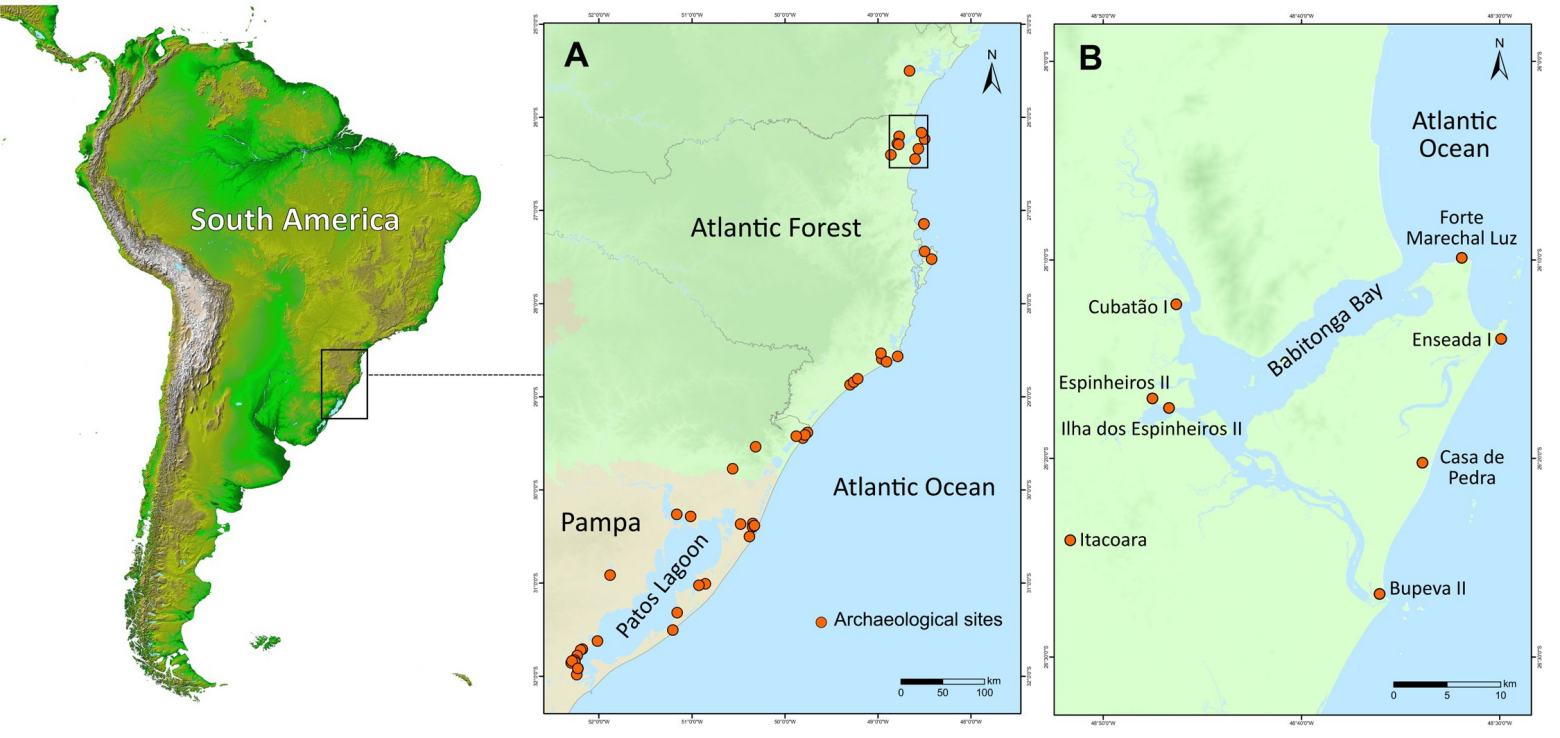
(A) Archaeological sites with fish remains, and (B) archaeological sites with fish remains in Babitonga Bay. Maps generated using ArcGIS 10.7 ([42]), CGIAR Consortium for Spatial Information ([43]) and NASA/JPL-Caltech (adapted from https://www.jpl.nasa.gov/images/pia03388-south-america-shaded-relief-and-colored-height).

This work presents an extensive review of the published data on marine and freshwater fish stocks exploited by Middle and Late Holocene Indigenous coastal populations in southern Brazil (Fig 1A). We assessed species composition and relative abundances through space and time, and compared fish functional traits (trophic level, maximum body size and maximum body mass) between archaeological and modern catches. We show that socially and economically important species for present day small-scale and industrial fisheries were extensively targeted by pre-European Indigenous groups. Narrowing our analysis to sites in Babitonga Bay, one of the largest estuarine systems in the Southern Atlantic and the region with the largest concentration of pre-European archaeological sites in coastal Brazil (Fig 1B), our study revealed that species of high trophic level and large body size and body mass were commonly exploited in the past, suggesting they were more abundant and easily encountered. Only in recent times have fisheries moved to small-bodied and lower trophic level species. We hypothesise that increasing fishing efforts and other coastal stressors have contributed to the decline in abundance of some of these high trophic level and large bodied species in modern fisheries. Our study offers a pre-market baseline to assess changes in species abundance, composition and function through time that is currently absent in one of the largest fish producing regions of the South Atlantic.

### Environmental and archaeological setting

The study area is located between 25°S and 31.5°S latitude and encompasses nearly 1000 km of coastline between the southern Atlantic Forest and the grassland Pampa biomes. The region includes several ecosystems supporting a great diversity of fish [17], including stocks of economic importance to both small-scale and industrial fisheries [25–27], such as Sciaenidae (e.g. *Micropogonias furnieri*, *Pogonia courbina*), Ariidae (*Genidens* sp.), Mugilidae (*Mugil liza*, *Mugil curema*), Paralichthyidae (*Paralichthys* sp.) and Pomatomidae (*Pomatomus saltatrix*), among others, as well as several species of sharks and rays [26, 27]. Most of these ecosystems evolved to their modern configurations during the Late Holocene [44–47]. Currently, the southernmost sector extends from Patos Lagoon (31.5°S) to the Santa Marta Cape (28.6°S) and is characterised by a wide continental shelf (120 to 230 km), with a gentle slope. The NE structure of the basement, subparallel to the coast, favoured the presence of continuous beaches of hundreds of kilometres and the development of the most extensive pairs of sandy barriers and lagoons in Brazil [48, 49]. This area includes large choked lagoons, such as Patos Lagoon, with salt marshes occurring in most tidal flats. Moving north, the change in coastline orientation favours coastal upwelling [50], and mangrove systems become dominant. From the Laguna lagoonal system (Mirim, Imaruí, and Santo Antônio lagoons) (28.1°S) to Babitonga Bay (26.7°S), the coastal sector has a narrower and steeper inner shelf and a more restricted coastal plain than neighbouring sectors, with headlands, rocky shores, pocket beaches and small embayments [49]. The coastal sector north of Babitonga Bay and adjacent to Santos Basin has a gentle slope, dominated by beaches over ten kilometres long with NE orientation, mostly separated by relatively small headlands and wide estuaries, such as the bays of Laranjeiras, Guaratuba and Paranaguá.

Estuaries and coastal lagoons were of primary importance for Indigenous populations in the study area before and during the earliest phases of contact with Europeans in the 16th century AD [51]. Marine resources were also exploited in a range of coastal habitats, including rocky shores [52] and oceanic islands [53], with considerable chronological and cultural variability. The earliest evidence of marine fish exploitation is associated with the Umbu cultural tradition, beginning around 9000 years ago [54, 55]. Contemporary to these groups, other populations depended largely on marine resources and raised monumental shell mounds locally known as *sambaquis* between 7000 and 500 years ago [56]. Along the Pampa biome and the La Plata basin in southern Brazil, Uruguay, and Argentina, groups known as *Cerritos* exploited fish and wetland resources between 4700 and 200 years ago [57]. From 1200 to 500 years ago, groups who produced ceramic artefacts attributed to the Taquara-Itararé cultural tradition also exploited fish as the main source of dietary protein in the southern Atlantic Forest coast [58, 59]. Finally, Tupiguarani and Guarani groups who settled in these coastal areas from around 1000 years ago until European contact [60], complemented their plant-based economies with fish from coastal environments [61].

## Material and methods

### Literature survey and data compilation

Faunal information was obtained from 71 reports produced between 1975 and 2022 on faunal assemblages recovered from Middle and Late Holocene sites along the southern coast of Brazil (S1 and S2 Tables). We limited our review to coastal sites, using a maximum distance from the current shoreline of 100 km, following Small and Nicholls [62]. This was also motivated by the fact that the high relative position of the sea level (*ca*. +3 m 5000 years ago [45, 63]) shifted the coastline further inland in areas with gentle slopes. Faunal reports included research articles (55%), academic dissertations and theses (38%), and book chapters (7%) available as physical and electronic copies in institutional repositories (universities, museums, public libraries) and publishers’ websites.

Reports were categorised according to qualitative and quantitative criteria proposed by Fossile et al. [64]: *Source A* (qualitative-quantitative)—presented detailed taxonomic identifications, and absolute and relative abundance for all taxa (Number of Identified Specimens (NISP) and/or Minimum Number of Individuals (MNI)); *Source B* (semi-quantitative)—presented detailed taxonomic identifications, and absolute and relative abundance for selected taxa; *Source C* (qualitative)—presented taxonomic identification with no quantitative information (S1 Table). Of the 71 documents listing fish remains, *Source A* (qualitative-quantitative), *Source B* (semi-quantitative) and *Source C* (qualitative) accounted for 60.6% (n = 43), 26.8% (n = 19) and 12.6% (n = 9), respectively. Data in sources A and B were used to calculate and compare relative taxonomic abundances within and among faunal collections, while data in sources A, B and C were used to derive species richness and their relative frequency distribution among sites. Whenever possible, taxonomic information was recorded to the species level, but for most sites only genus, and often classes, orders, and/or families were available. The nomenclature and ecological attributions follow WoRMS [65] and Eschmeyer’s Catalog of Fishes [66] (S3 and S4 Tables). Species richness (SR) was calculated using the Minimal Level of Taxonomic Identification, considering only the minimum hierarchical level for particular taxa. The conservation status of species was compiled from the IUCN Red List of Threatened Species [67] and the updated Red List of Threatened Species of the Chico Mendes Institute for Biodiversity Conservation (*Instituto Chico Mendes de Conservação da Biodiversidade*—ICMBio) [68]. The socioeconomic importance of species was compiled from the National Action Plan for the Conservation of Endangered Species and of Socioeconomic Importance in the Mangrove Ecosystem—PAN Mangrove [69].

We used NISP values and, in a few cases, MNI (Espinheiros II, RS-LS-11 and Itapoã; S4 Table) to express the absolute and relative abundances of taxa. NISP was reported in 77.4% of the analysed sites with fish remains, while MNI (with no corresponding NISP data) was reported in 5.7% of the sites. For sites where faunal assemblages were analysed for distinct areas, the absolute abundance of each taxa was aggregated. In the case of faunal assemblages that were published more than once, the most detailed study in both taxonomic and quantitative terms was considered. Total NISP values include all identified remains regardless of their taxonomic levels (from class to species). However, relative abundance of taxa was performed using NISP values after removing the number of remains generically identified as Actinopterygii (bony fish) and Elasmobranchii (cartilaginous fish). Trophic levels of exploited organisms (excluding class level and above) were attributed according to FishBase [70]. For order, family and genus we used the average values of species present in archaeological records in the region, or the average values of the species reported in the Brazilian Biodiversity Information System (SiBBr) [71]. One-way Analysis of Variance (ANOVA) followed by Tukey’s HSD tests (stats package in R) was used for comparing NISP and SR according to excavation mesh size (95% family-wise confidence interval). A Pearson correlation coefficient (stats package in R) was employed for measuring linear correlations between SR and NISP.

### Cultural and chronological assignments of pre-European fish assemblages

Faunal assemblages were compiled by archaeological sites taking into account their cultural phases and radiocarbon dates (calibrated years before present, cal BP). Cultural phases consist of well-established “traditions” based on site typology (e.g. shell mounds, earth mounds), the presence and type of key artefacts (e.g. stone tools, ceramics), and their “absolute” chronology based on radiocarbon dates. The latter allows the general assignment of cultural phases to the formal subdivisions of the Holocene based on natural climatic/environmental events (Early, Middle and Late) [72]. For example, faunal assemblages from Enseada I were separated by two distinct cultural phases including a Sambaqui (4050 cal BP) phase and a Taquara-Itararé (1050 cal BP) phase, both dated to the Late Holocene [73]. Fauna from the site of Sangão, instead, were computed separately for Early (8950 cal BP) and Late (4650 cal BP) Holocene occupations [54].

Radiocarbon dates for the analysed sites were obtained from the Brazilian Radiocarbon Database [74], on dates generated from a range of archaeological materials (marine shells, human and faunal bones, charcoal). Conventional radiocarbon dates were calibrated and modelled using OxCal v. 4.4 [75]. For sites with multiple dates (e.g. Forte Marechal Luz, Cubatão I, Jabuticabeira II, RS-PSG-07), the conventional dates were summed (Sum function) according to main cultural attributes, for example by grouping dates obtained from Sambaqui occupations, or from layers with ceramic artefacts of Taquara-Itararé tradition. In doing so, we estimated the median age of a particular “cultural” occupation (group median). Terrestrial samples were calibrated using the 100% atmospheric calibration curve for the southern hemisphere, SHCal20 [76]. Marine organisms were calibrated using the 100% Marine20 curve [77], applying an estimated average local marine radiocarbon reservoir correction value (ΔR) of -126 ± 29 for the coasts of São Paulo, Paraná, Santa Catarina and Rio Grande do Sul, generated from eight reference points between latitudes 32.0°S and 23.7°S [78–80], according to the Marine Reservoir Correction database. Given the high contribution of marine carbon to bone collagen of human individuals in this region, the radiocarbon dates on human bone collagen were modelled using a mixed curve (SHCal20 and Local Marine curve) adopting the same ΔR value reported above. We considered the average relative contribution of marine carbon to collagen of 52 ± 9%, which is the average estimated contribution recently obtained from dozens of human individuals from archaeological sites in Babitonga Bay [40]. Calibrated and modelled radiocarbon dates were rounded to 50 years (S5 Table).

### Comparing fish traits across time periods in Babitonga Bay

Fish data were compiled for three distinct chronological periods (and cultural phases) in Babitonga Bay: 4500–1150 cal BP (Sambaqui), 1050–600 cal BP (Taquara-Itararé), and AD 1994–2015 (modern fisheries). Fish assemblages dated to 4500–1150 cal BP were recovered from the Sambaqui phases of the sites Cubatão, Espinheiros II, Ilha dos Espinheiros II, Forte Marechal Luz, Enseada I, Bupeva II and Itacoara; fish assemblages dated to 1050–600 cal BP included the Taquara-Itararé phases documented in the sites of Forte Marechal Luz, Enseada I, Bupeva II and Itacoara (Fig 1B); modern fish assemblage composition (241 species) were obtained from surveys conducted in Babitonga Bay from AD 1994 to 2015 [81, 82] (S6 and S7 Tables). A total of 124 species were documented as fisheries targets for Babitonga Bay across the three studied periods. Of these, 62 species were recorded for the Sambaqui cultural phase, 34 for the Taquara-Itararé cultural phase [41, 73, 83–86], and 94 for the modern period (*Projeto de Monitoramento da Atividade Pesqueira em Santa Catarina*—PMAP/SC, available at: http://pmap-sc.acad.univali.br/index.html) (S6 and S8 Tables).

For each species recorded, we compiled trophic level, trophic group, maximum body size (cm) and maximum body mass (g). Fish trophic level was taken from FishBase (see above) and ranged between 2.0 to 4.9. Trophic group categories were compiled from Quimbayo et al. [87] and complemented with information from other literature [88–107]. The categories were invertivores, herbivores, macrocarnivores, omnivores, piscivores and planktivores. Maximum body size and body mass were also obtained from Quimbayo et al. [87] and from other literature [70, 108–111]. Fish species were categorised into body size classes based on their maximum body sizes. These categories were: <7 cm, 7–15.0 cm, 15.1–30.0 cm, 30.1–50.0 cm, 50.1–80.0 cm, and >80 cm. Body size categories and trophic groups were combined for each species to define fish functional entities (FEs).

Differences in fish species’ trophic levels, maximum body sizes, and maximum body masses were assessed using a null model approach, under which the observed traits in each period (4500–1150 cal BP, 1050–600 cal BP and AD 1994–2015) were contrasted against null values obtained from randomly sampling the total species pool [112]. The total species pool corresponds to all fish taxa caught across the different study periods (n = 124) combined with those that were not targeted but compose the regional fish assemblages (n = 241). This total pool (n = 365) is, therefore, equivalent to the total species richness and trait variability in the region, regardless of the time period. For each trait and time period, we contrasted the distribution of observed values with the distribution of 1000 random samples of the total pool. The observed trait values were compared to those of random assemblages (null values) for each time period using two-sided t-tests. Box plots and violin plots were used to explore the distribution and differences between observed and null values. To test for differences in fish traits caught across time periods we used One-way Analysis of Variance (ANOVA) followed by Tukey’s HSD tests. Fish maximum body size and maximum body mass were positively and significantly correlated (r^2^ = 0.67; *p* < 0.005), and log-transformed before analysis. We opted to maintain both traits in our analysis to explore differences across periods (S1 Text).

We further explored differences in the proportion of fish species belonging to body size categories, trophic group categories, as well as functional entities (FEs) across time periods. These would indicate whether certain body sizes, fish trophic groups and functional groups corresponded to a greater proportion of fisheries targets in particular time periods. Also, such analysis can reveal changes and fisheries characteristics across time. To test the statistical significance in the proportions of fish body sizes and trophic categories across time periods we used a two-tailed Binomial test (*p* < 0.05). All analyses were conducted in R Studio Software [113].

Finally, the data presented is part of the Brazilian Zooarch Database (ZooarchBR) and is stored at the Brazilian Biodiversity Information System (*Sistema de Informação sobre a Biodiversidade Brasileira*—SiBBr) [71]. SiBBr consists of a set of data and information on Brazilian biodiversity and ecosystems linked with the Global Biodiversity Information Facility (GBIF), providing subsidies for government management related to conservation and sustainable use [71]. This platform is developed by the Brazilian Ministry of Science, Technology and Innovation (*Ministério da Ciência*, *Tecnologia e Inovações*—MCTI), with technical support from ONU Environment (UNEP) and financial support from the Global Environment Facility (GEF). The data presented here is registered in the SiBBr in the category Occurrence Records (inventories and/or research projects) through the TRADITION Project, following the Darwin Core (DwC), an international standard recognized by the scientific community for metadata. The data is also available in https://doi.org/10.5281/zenodo.7925975.

## Results

### Quantitative and qualitative data assessment

Faunal information (marine, freshwater and terrestrial animals) was compiled for 374 archaeological sites (71 reports) distributed along the southern Atlantic Forest (89%) and the Pampa (11%) biomes, from publications produced over the last 47 years (Fig 1 and S1 and S2 Tables). Fish remains were reported in 53 sites (14.4%, sources A, B and C) (S3 Table). Of these, 44 sites (83%, sources A and B) contained information on the absolute and relative abundance of taxa (S4 Table). Recovery and analytical techniques differed considerably among sites. For example, of the 44 sites with the absolute and relative abundance of taxa, fish remains were retrieved through sieving sediments in 73% (n = 32), but using distinct mesh sizes: 2 mm (31.2%, n = 10 sites), 3 mm (31.2%, n = 10 sites), 4 mm (9.4%, n = 3 sites) and 5 mm (28.1%, n = 9 sites). The volume of sediment was reported, or could be estimated from excavated areas, for 84% of the sites with fish remains (n = 37), and ranged from 0.06 to 13.8 m^3^. In the majority of sites (98%, n = 43) both bones and otoliths were used for specimen quantification (NISP), while in 80% of sites (n = 35) otoliths and bones were used for taxonomic identification (S4 Table).

**Fig 2:**
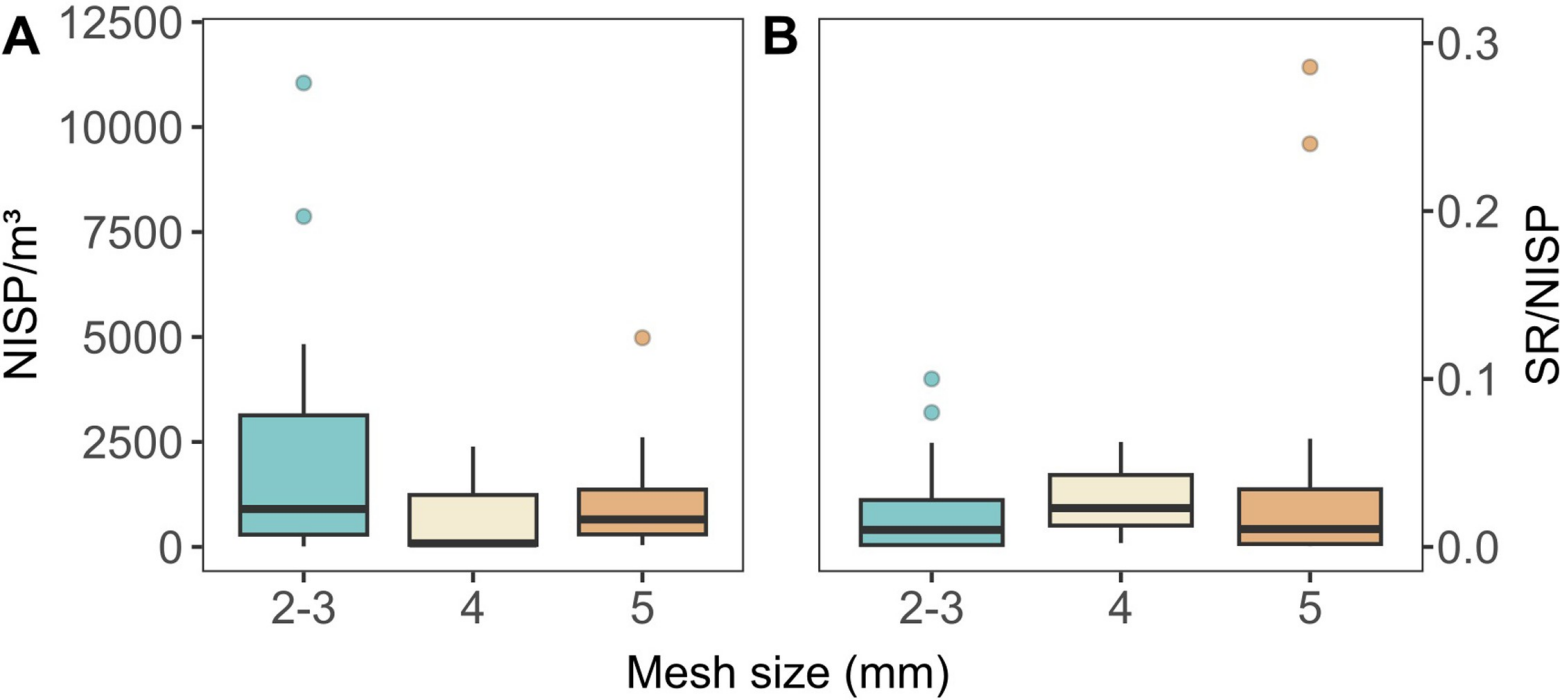
Boxplot showing the distribution of A) NISP/m^3^ versus mesh size and B) SR/NISP versus mesh size.

Archaeological assemblages in sources A and B were dominated by bony fish (total NISP = 876,622; 98.8% of total remains), followed by cartilaginous fish (total NISP = 10,603; 1.2%). Among bony fish, 17% of the remains could be identified beyond the class level (Actinopterygii), while for cartilaginous fish 56.7% were identified beyond the Elasmobranchii class. These proportions remained substantially comparable across the sites, regardless of their geographic location and chronology. When considering the Minimum Level of Taxonomic Identification in sources A, B and C, 109 taxa were reported, of which 79 belonged to bony fish and 30 to cartilaginous fish (S3 Table). Differences in recovery and analytical methods (sometimes referred to as second-order changes [114]) can dramatically affect fundamental and derived measurements of faunal remains such as the taxonomic abundance (NISP) and richness (SR) [114–116]. Here we explored the effect of sieving with distinct mesh sizes (2–3 mm, 4 mm and 5 mm) on both total NISP and SR (Fig 2A and 2B). For this purpose we combined data from 2 and 3 mm (2–3 mm) mesh because both are considered adequate for the recovery of fish remains [117, 118]. NISP was normalised for the volume of sediment (NISP/m^3^) to account for additional size-effect. SR was also normalised for contextual total NISP values (SR/NISP). Surprisingly, the results reveal no significant statistical differences between NISP/m^3^ produced by using 2–3 and 4 mm mesh (*p* = 0.2936), nor between 2–3 and 5 mm mesh (*p* = 0.6817), or 4 and 5 mm mesh (*p* = 0.6032). Similarly, derived SR/NISP were statistically indistinguishable between 2–3 and 4 mm mesh (*p* = 0.6902), and between 2–3 and 5 mm mesh (*p* = 0.5218). Again, SR produced through the use of 4 and 5 mm mesh were statistically comparable (*p* = 0.9796).

**Fig 3:**
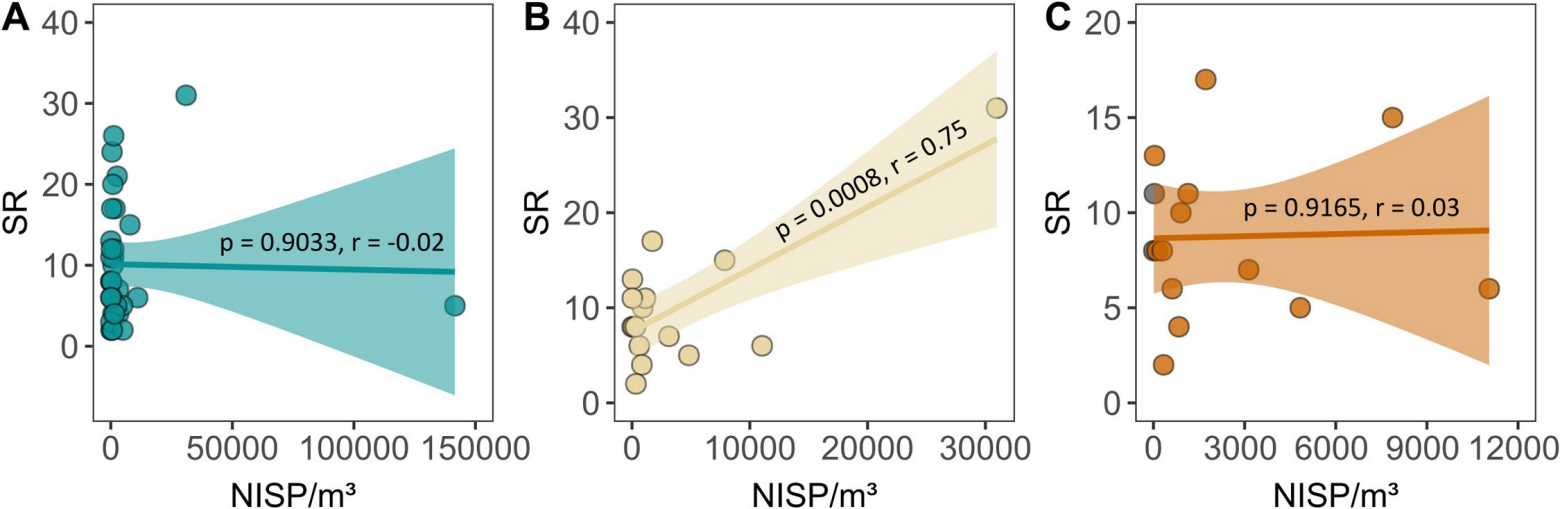
Correlation between SR and NISP/m^3^ for A) the entire dataset (sources A and B), B) faunal remains recovered with 2–3 mm mesh size and identified using both bones and otoliths, and C) the same data in B after removing a single outlier (site of *Rio do Meio*) with the highest SR and NISP/m^3^.

We also assessed the sample size-effect by comparing SR with NISP/m^3^ [115, 116], under the theoretical proposition that, all things being equal, SR positively correlates with NISP/m^3^. The results revealed no significant correlations (*p* = 0.9033, r = -0.02, n = 28) between SR and NISP/m^3^ for the entire dataset (sources A and B) including distinct recovery (all mesh sizes) and identification (otoliths and/or bones) methods (Fig 3A). By contrast, when selecting data associated with 2–3 mm mesh size and identified using both bones and otoliths, a significant positive correlation (*p* = 0.0008, r = 0.75, n = 14) emerged between SR and NISP/m^3^ (Fig 3B). This correlation, however, depended on a single endmember with the highest SR and NISP/m^3^ (site of *Rio do Meio*), which when removed dissolved the positive correlation (*p* = 0.9165, r = 0.03, n = 13) (Fig 3C). In conclusion, although distinct recovery techniques may have affected the quality of the data in terms of SR and taxonomic abundances, the magnitude of these second-order changes remains unclear.

### Pre-European fish compositions

**Fig 4:**
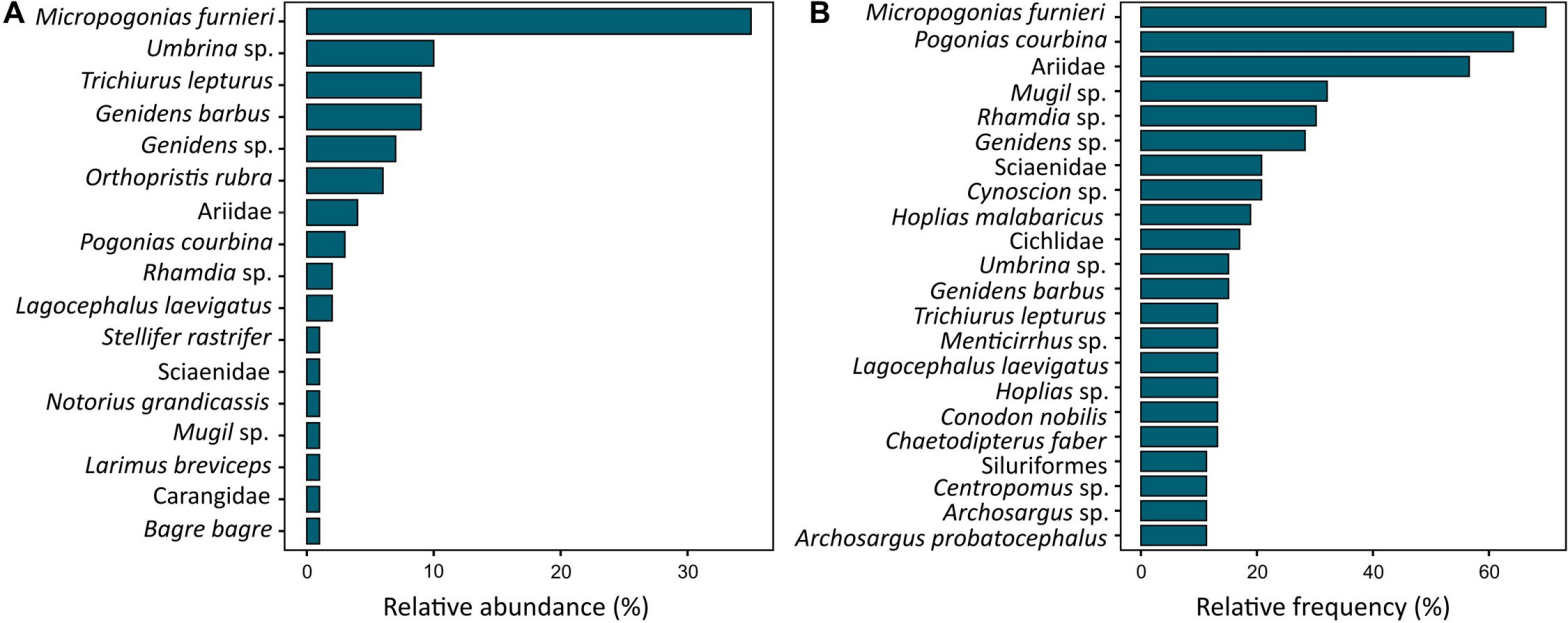
(A) Relative abundance of bony fish taxa above 1% in pre-European assemblages, and (B) relative frequency of bony fish taxa above 10%.

**Fig 5:**
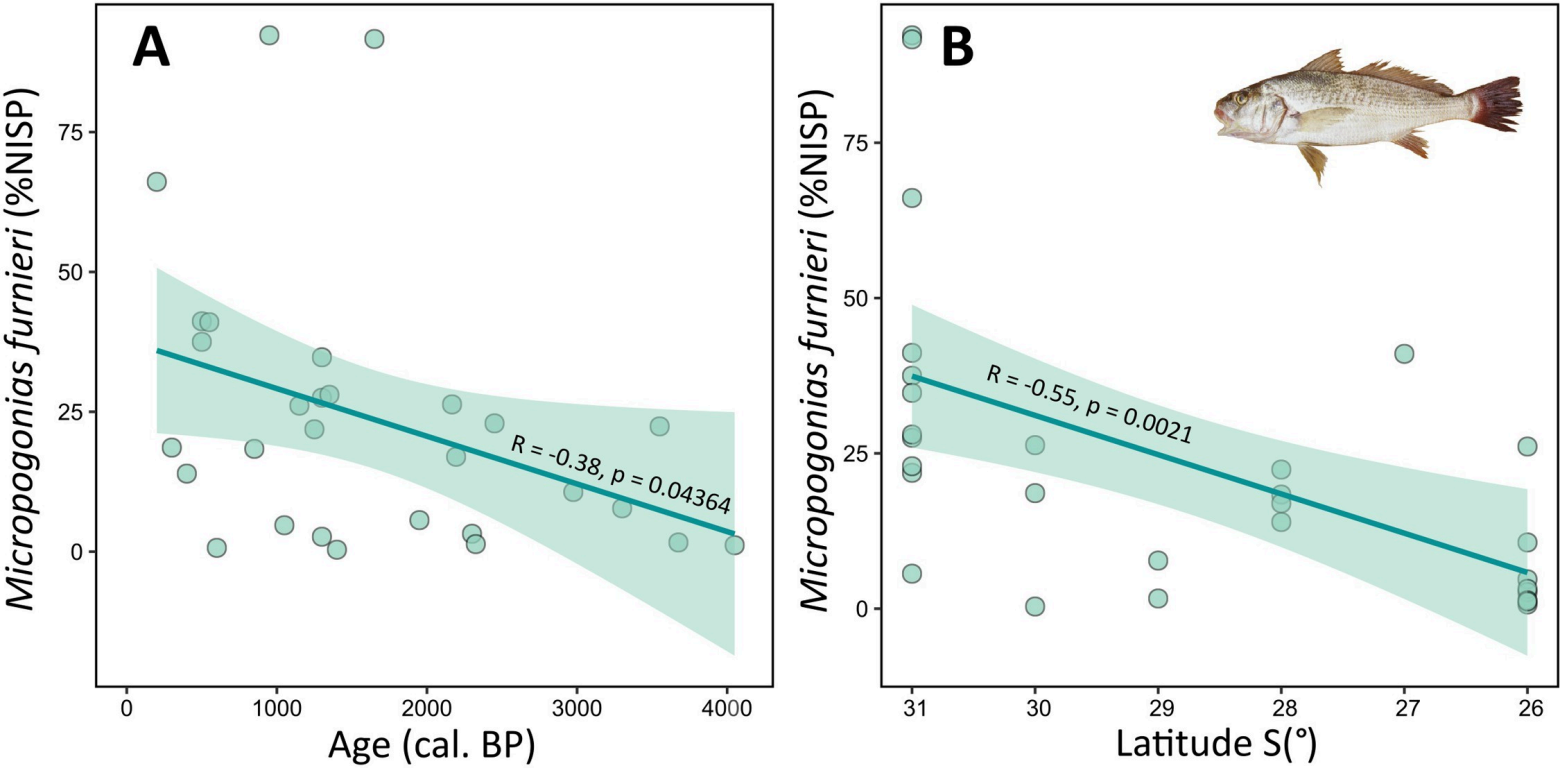
Relative abundance of *Micropogonias furnieri* as a function of (A) the median radiocarbon age of sites (modelled cal BP), and (B) latitude in pre-European assemblages. Green band represents the 95% confidence interval.

Sites with fish remains had median calibrated dates mostly concentrated between 5402 and 200 cal BP. The only exceptions are the Early Holocene deposits of Sangão, which had two modelled median radiocarbon dates of 8150 and 9750 cal BP (S5 Table). Among bony fish species belonging to Sciaenidae (e.g. *Micropogonias furnieri*, *Umbrina* sp., *Pogonias courbina*) and Ariidae (e.g. *Genidens barbus*, *Bagre bagre*, *Notarius grandicassis*) accounted for *ca*. 50% and 23% of the identified remains, respectively (Fig 4A). They were also widely distributed in the archaeological record, with remains of Ariidae reported in 92% (n = 49) of the sites, and Sciaenidae in 79% (n = 42) (Fig 4B). At the species level, the marine-brackish, demersal and oceanodromous *Micropogonias furnieri* (whitemouth croaker) emerged as the most abundant and broadly captured species, representing 33% (NISP = 50,327) of the identified remains, and occurring in 70% (n = 37) of the archaeological sites (Fig 5A). Its capture appears to have increased significantly with time, most notably from *ca*. 2000 cal BP (Fig 5B) around Patos Lagoon (*ca*. 31°S), a key reproductive and feeding ground for this species in southern Brazil [26].

**Fig 6:**
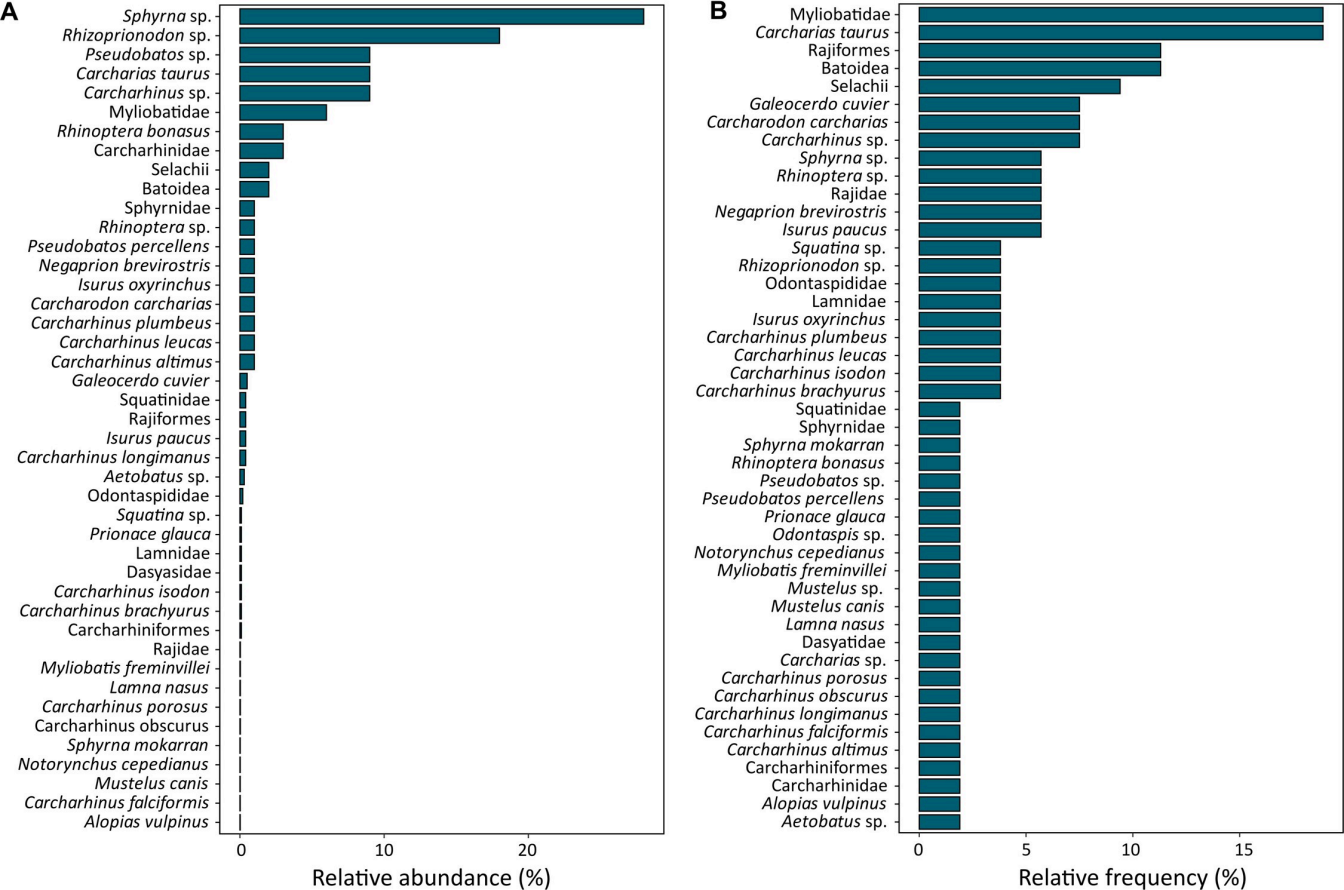
A) Relative abundance and B) relative frequency of cartilaginous fish taxa in pre-European assemblages.

In terms of relative frequency, *Micropogonias furnieri* was followed by *Pogonias courbina* in *ca*. 64% of sites (n = 34), and unidentified Ariidae species (57%), *Mugil* sp. (32%), *Rhamdia* sp. (30%) and *Genidens* sp. (28%). With the exception of *Rhamdia* sp. (freshwater catfish), the taxa reported above are commonly distributed in near-shore coastal waters, including estuaries, tidal flats and mangrove systems [82]. Freshwater fish contributed to 0.6% of the remains, and were represented by *Rhamdia* sp., *Hoplias malabaricus*, *Hypostomus* sp., and *Synbranchus marmoratus* (S4 Table). Cartilaginous fish were mostly represented by remains of *Sphyrna* sp. (28.3%) and *Rhizoprionodon* sp. (18.5%), followed by *Carcharias taurus* (9.5%), *Carcharhinus* sp. (9.3%), *Pseudobatos* sp. (8.9%), Myliobatidae (5.6%) and others (Fig 6A). Overall, sharks and rays occurred in 53% (n = 28) of the sites with fish remains. Of these, Myliobatidae (rays) and *Carcharias taurus* were reported in 19% (n = 10) of the sites, followed by unidentified rays (infraclass Batoidea and order Rajiformes) and sharks (Selachii) in 11.3% and 9.4%, respectively, and finally by several species including *Carcharhinus* sp., *Galeocerdo cuvier*, *Carcharodon carcharias*, *Rhinoptera* sp., and *Negaprion brevirostris* (Fig 6B). Currently, these species are reported along the southern coast of South America (e.g. *Carcharias taurus*, *Notorynchus cepedianus*, *Lamna nasus*), while others, such as *Negaprion brevirostris*, *Sphyrna mokarran* and *Carcharhinus porosus*, are not found or are less frequent in southern Brazil [119–121].

**Fig 7:**
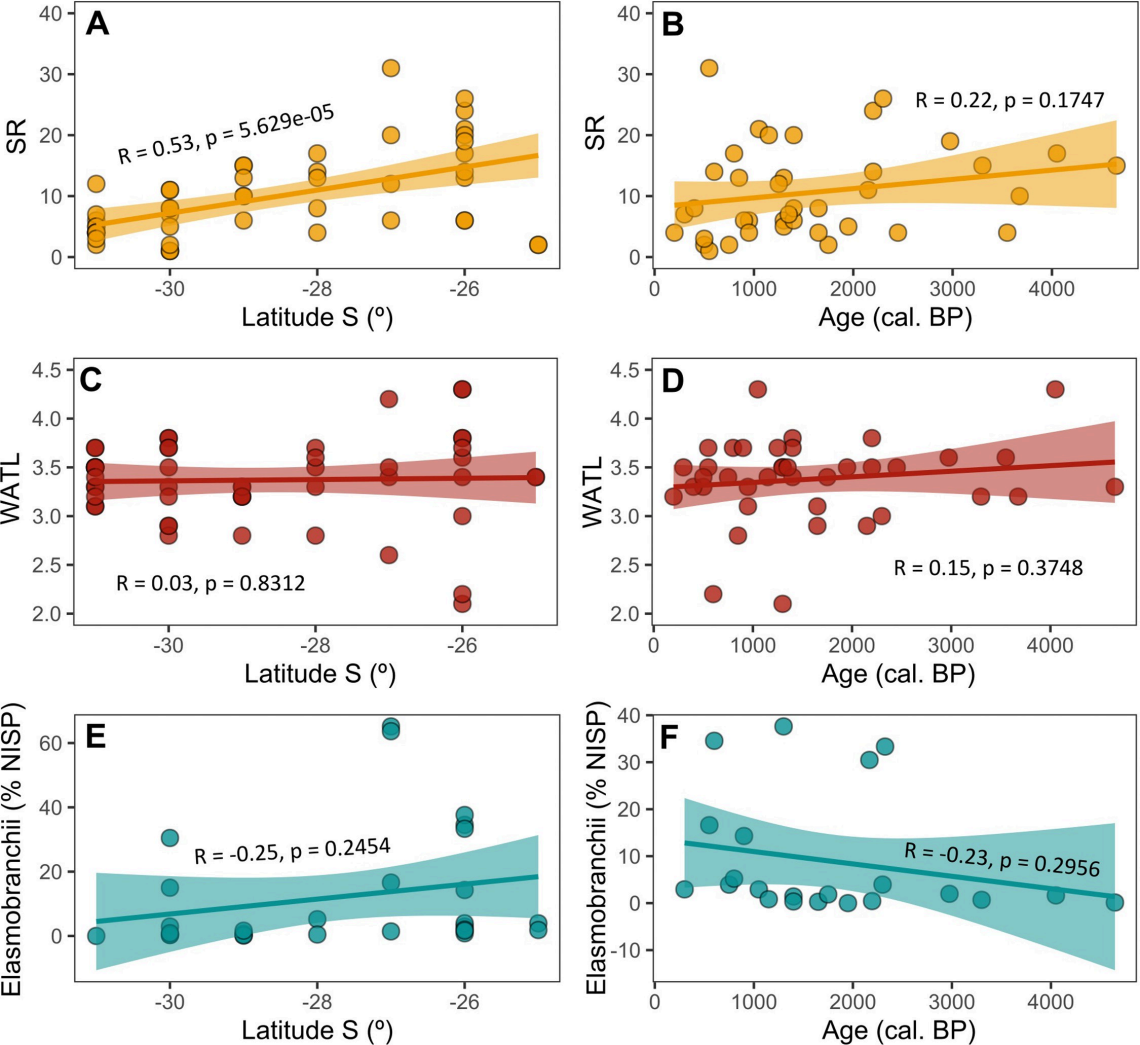
Species Richness as a function of A) the latitude and B) the median radiocarbon dates of the sites; C) weighted average trophic level per latitude and D) chronology; E) relative abundance of Elasmobranchii remains in relation to latitude and F) chronology of the sites. The bands represent the 95% confidence interval.

**Fig 8:**
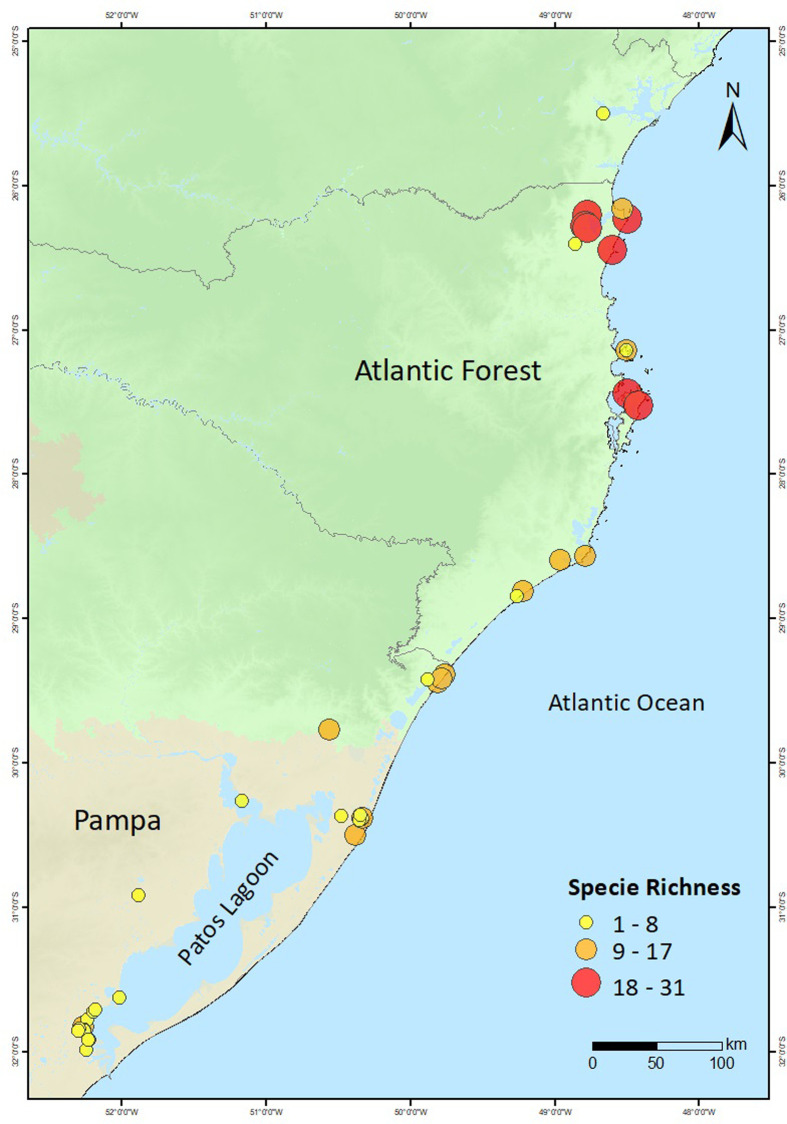
Latitudinal changes in species richness. Maps generated using ArcGIS 10.7 ([42]) and CGIAR Consortium for Spatial Information ([43]).

Bony and cartilaginous fish diversity significantly decreased southwards (Fig 7A), but remained stable over time (Fig 7B). The latitudinal gradient in species diversity is consistent with studies showing a similar pattern in modern fish communities in the southwestern Atlantic Ocean [122–124]. By contrast, the weighted average trophic level (WATL) of fish assemblages did not correlate with latitude (Fig 7C) nor with site chronology (Fig 7D). Interestingly, sharks and rays increased in sites postdating 2200 cal BP (Fig 7F), particularly in areas located between Babitonga Bay (26°S; Bupeva II, Forte Marechal Luz) and Santa Catarina Island (27°S; Rio do Meio) (Fig 7E). Even though their relative abundance was not significantly correlated with age or latitude, the results are consistent with stable isotope analyses of human bone collagen indicating that from *ca*. 2200 years ago groups in Babitonga Bay and north of Santa Catarina Island secured most of their dietary proteins from high trophic level fish species [40]. Cartilaginous fish also accounted for more than 60% of the fish remains at the sites of Rua 13 and Sambaqui do Papagaio, both located in the aforementioned region, but lacking absolute chronology. The presence of adult, juvenile and neonate specimens [58, 125] suggests that humans were exploiting sharks and rays in a range of habitats, including nursery areas such as mangrove systems [126], as documented in other areas further north [127]. While it is plausible that some shark teeth could indicate trading networks, it is important to note that they are consistently found alongside a significant quantity of shark vertebrae and other fish remains. This suggests that the presence of shark teeth in most sites reflects local fishing activities. Together, these results suggest that captures along the Pampa (31.5°S) and southern Atlantic Forest coasts (28°S) were generally less diverse compared to more northern sites (27 to 26°S), as in the northern Santa Catarina coast (Fig 8). However, as discussed above, the diversity and abundance of the catches must have been higher than reflected in the collected data. High fish diversity, incomplete reference collections and disparity in recovery techniques, elevated fragmentation and lack of diagnostic features in archaeological bones are some of the common challenges that prevent the successful identification and adequate quantitative representation of fish remains in the regional archaeological record.

### Trait variation of fish caught in Babitonga Bay across time periods

Babitonga Bay has the largest concentration of pre-European archaeological sites in coastal Brazil. Previous studies have indicated that high trophic level and large-bodied fish species were commonly targeted by Sambaqui (4500–1150 cal BP) and Taquara-Itararé (1050–600 cal BP) groups in pre-European times [40], which contrasts with modern catches reported by the *Projeto de Monitoramento da Atividade Pesqueira em Santa Catarina* ([81, 82] in the region. We tested this hypothesis by comparing fish functional traits for three distinct time periods (4500–1150 cal BP, 1050–600 cal BP and AD 1994–2015). We assessed differences in fish species trophic levels, maximum body sizes, and maximum body masses using a null model approach, under which the observed traits in each period were contrasted against null values obtained from randomly sampling the total species pool [112].

**Fig 9:**
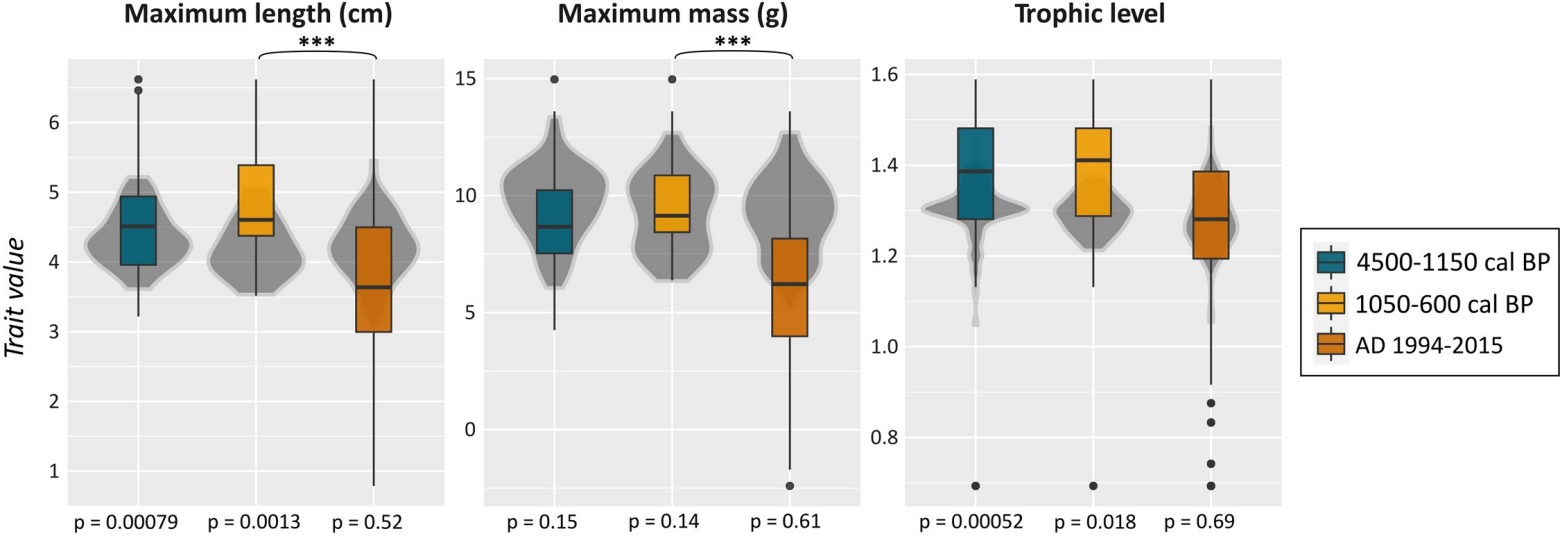
Fish traits exploited between 4500–1150 cal BP, 1050–600 cal BP and the modern period (1994–2015) for A) maximum body sizes, B) maximum body masses and C) trophic levels. Box plots represent observed values from fisheries catches across 4500–1150 cal BP, 1050–600 cal BP and the modern period (AD 1994–2015). Vertical black line denotes the median observed values, and black points are data outliers. Grey violin plots on the back represent the density distribution of null model values from random samples of the total species pool. P-values for pairwise comparisons of observed traits versus randomised data are shown below the boxes. Asterisks mark significant differences between time periods as indicated by Tukey HSD.

Our results suggest that catches in the periods of 4500–1150 cal BP and 1050–600 cal BP were composed of individuals of larger body size and body mass relative to modern fish catches (Fig 9A–9C). Between time periods, there were no significant differences in the trophic level of fish caught, but a slight decrease was detected in the minimum trophic level of fish species reported for the modern period (AD 1994–2015; MinTL = 2.9) compared to 1050–600 cal BP (MinTL = 3.3). Significant differences were instead only observed for the maximum body size and body mass of catches between 1050–600 cal BP and the modern period (*p* < 0.005 for both). Catches from 4500–1150 cal BP and 1050–600 cal BP were statistically indistinguishable for maximum body size and maximum body mass.

Within time periods, the maximum body sizes of fish in pre-European catches (time periods of 4500–1150 cal BP and 1050–600 cal BP) differed significantly from randomly generated assemblages (t-tests, *p* = 0.0007, samples from the total species pool), while modern catch (AD 1994–2015) body sizes were within those predicted at random (t-test, *p* = 0.52). The trophic levels of fish caught in periods 4500–1150 cal BP and 1050–600 cal BP were greater than that from the random assemblages (*p* = 0.00052 and 0.018 respectively); while the trophic level of species in modern catches did not differ from the random expectations (*p* = 0.69). For body mass, there were no significant differences from observed to null trait values (Fig 9A–9C).

**Fig 10:**
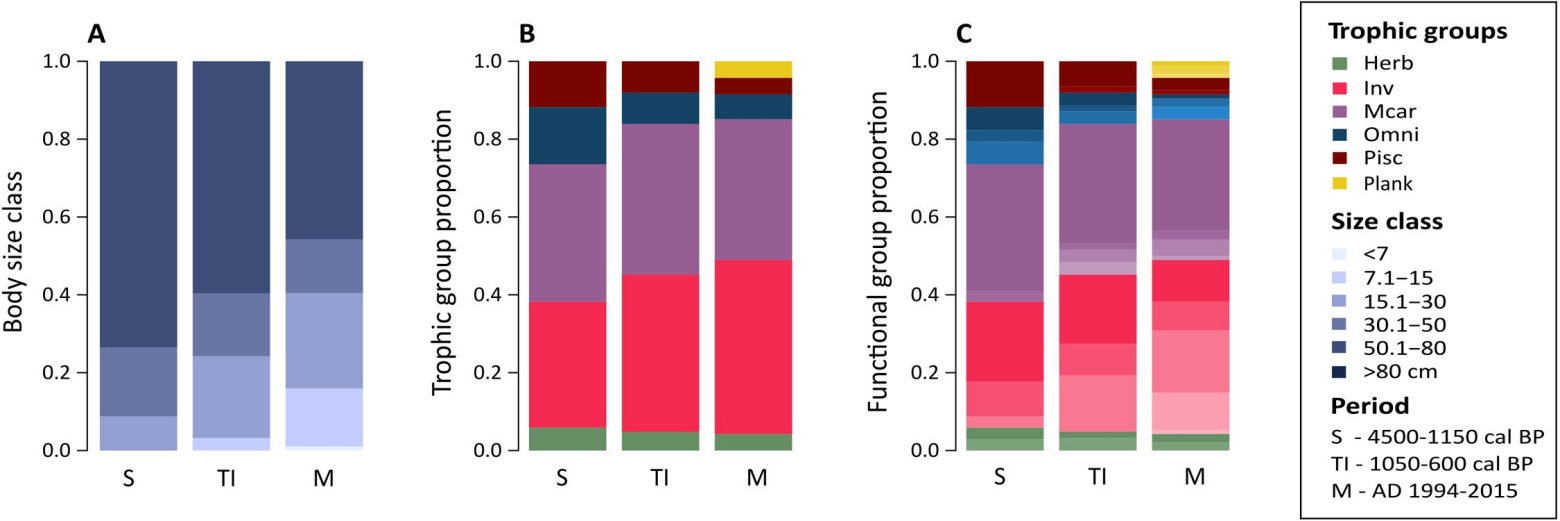
Fish traits and functional groups across time periods. A) The proportion of fish species within different body size categories, B) trophic groups, and C) functional entities between pre-European (4500–1150 cal BP and 1050–600 cal BP) and modern (AD 1994–2015) periods. In C, functional entities represent the combination of fish trophic groups and body size classes, and darker tones represent larger body size categories within the same trophic group. Two-tailed Binomial tests reveal significant (*p* < 0.05) differences in the proportions of 15.1–30 cm species between the 4500–1150 cal BP and modern (AD 1994–2015) periods.

Overall, the composition of captured species was dominated by large-bodied fish (> 50 cm) throughout the studied periods (4500–1150 cal BP ∼90%; 1050–600 cal BP ∼76% and modern ∼58% of species). Modern populations included species with a broader range of body size categories, varying from fish < 15 cm in total length to large bodied species > 80 cm (Fig 10A). Fish maximum body sizes ranging from 15.1 to 30 cm corresponded to 14% of the species in modern catches, which was significantly different (*p* < 0.001) to catches in the period 4500–1150 cal BP, with only 3.2% of species identified (*Cathorops spixii* and *Isopisthus parvipinnis*) within this body size category. Medium and small-bodied fish species corresponded to a minor proportion of catches in pre-European times (3–20% for species up to 50 cm total length). Species with total length > 80 cm accounted for 45.7% of modern species composition, while they represented 59.6% and 73.5% of catches dated to 4500–1150 cal BP and 1050–600 cal BP, respectively (e.g. *Trichiurus lepturus*, *Caranx latus*).

Compared to modern assemblages, pre-European catches were characterised by a greater contribution of piscivore and omnivore species (Fig 10B). Piscivores accounted for 8.1% to 11.7% of species in fish remains from periods 4500–1150 cal BP and 1050–600 cal BP, respectively (e.g. *Bagre bagre*, *Pomatomus saltatrix*, *Trichiurus lepturus*), compared to 4.2% in modern assemblages. The proportion of macrocarnivores was similar across all periods (∼35% of species). The contribution of invertivore fish slightly increased across periods, accounting for 40.3% and 32.3% in catches from periods 4500–1150 cal BP and 1050–600 cal BP (e.g. *Genidens barbus*, *Lagocephalus laevigatus*, *Micropogonias furnieri*), respectively, and reaching up to 44.6% in the modern period (AD 1994–2015). Planktivores were only documented in modern assemblages (4.2%). Finally, the proportion of fish functional entities reveals that it is the addition of fish within larger body size classes that greatly characterises pre-European catches (Fig 10C). Modern assemblages have a much greater contribution of small and medium-sized invertivores, as well as a lower contribution of large bodied (> 80 cm) piscivore species. While in catches from the period 4500–1150 cal BP, macrocarnivores were mostly large bodied (50.1–80 cm and > 80 cm), in catches from 1050–600 cal BP and in modern assemblages (AD 1994–2015) there is an increase of smaller macrocarnivore species (15.1–30 cm and 30.1–50 cm).

## Discussion

### Variations in pre-European fish compositions

Our review of the literature shows that several species of high social and economic value today were exploited by coastal societies in southern Brazil over the last 9500 years [128–130]. We acknowledge that the discrepancies in recovery and analytical techniques among reports complicate attempts to interpret data across distinct sites, and over spatial and temporal scales. Moreover, the faunal record presented here reflects dietary preferences, food processing, site function (e.g. cemeteries, residences), symbolic and ritual practices, and taphonomic factors that may have varied in time and space. Nevertheless, archaeology remains one of the few sources of information available to elucidate species distribution and relative abundance in the past, and particularly in data-deficient countries such as Brazil. Even though the relative abundance and distribution of species in archaeological contexts may be distorted due to the combination of first-order and second-order changes to the archaeological record (see [114]), the zooarchaeological data produced over the last 47 years in Brazil offer a unique approximation of which species were targeted in this region over time scales surpassing those of modern observations. Integrating methodologies such as geometric morphometrics, stable isotopes, archaeogenomics, and proteomics in neotropical zooarchaeology studies can significantly enhance its relevance to marine conservation.

The diversity of the catches are similar to those reported for modern small-scale commercial landings in the region [131–133], even though only 8% (n = 13) of the species accounted for the majority of the identified fish remains (NISP > 1%). Of these, a few demersal and estuarine-dependent species belonging to Sciaenidae (e.g. *Micropogonias furnieri*, *Umbrina* sp., *Pogonias courbina*) and Ariidae (e.g. *Genidens barbus*, *Bagre bagre*, *Notarius grandicassis*) were predominantly targeted in the past. They possibly formed large populations in coastal lagoons and estuaries, providing humans with an abundant and reliable supply of marine proteins year-round [39, 40].

Several lines of evidence indicate that pre-European coastal communities were able to extract volumes of fish comparable to or greater than subsistence catches in recent times [41]. Stable isotopes analyses also revealed that fish consumption per capita (contribution of marine protein to individual diet) was higher among pre-European communities [40] compared to local modern populations in coastal Brazil [134–136]. Nevertheless, the archaeological faunal record does not provide evidence for measurable human impacts on coastal organisms in this region. While most of these groups exploited crucial habitats for species reproduction and conservation (mangroves, salt marshes [126]), and targeted juvenile individuals in nursery grounds (e.g. sharks [137]), compelling evidence for ecological impacts are still lacking. If such detrimental impacts occurred, they were likely limited or localised, possibly permitting stock recovery over relatively short periods of time. For example, *Micropogonias furnieri* dominated fish assemblages in Patos Lagoon, with catches increasing in the last 2000 years in relation to human population growth [138] and stabilisation of environmental conditions, notably sea level, in the region [47]. Yet, there is no evidence that this species was affected by overfishing, such as could be indicated by a declining number in catches [139, 140]. By contrast, the targeting of *M*. *furnieri* by small-scale and industrial fisheries in recent times have led to a decrease in abundance to the point that it is now considered overexploited [25, 26]. However, further studies are required for conclusive interpretations to be drawn.

It is particularly striking that *Mugil* sp., despite occurring in 32% of sites with fish remains, represented only 0.7% of combined NISP values (excluding remains generically identified as Actinopterygii and Elasmobranchii). At least one species of Mugilidae (*Mugil liza*) is abundantly captured by communities along the southeastern coast of southern Brazil during austral autumn and winter, from May to July, and constitutes a cultural seasonal keystone seafood in this region [141]. The artisanal catches are obtained through the use of distinct fishing gear, but large catches are mostly secured by means of beach seine [142]. It could be argued that the relatively low abundance of *Mugil* sp. in pre-European catches reflect a lack of mass-capture fishing technology, such as large nets. Archaeological evidence for the use of fishing nets has been found in this region, such as stone sinkers and weights [143], as well as plant-based cordage and other artefacts [144], but their effectiveness for capturing large schools of Mugilidae remains a matter of debate. Significantly, Indigenous culinary practices described in some 16th century European chronicles provide some insight into the complexity of the taphonomic processes potentially affecting fish assemblages in this region. For example, Hans Staden in the first half of the 16th century reported that Guarani groups of the southern coast of Brazil processed fish (possibly Mugilidae) to make flour [145]. Although the origin and details of such practices are unknown, fish drying and grinding would imply that some species may have undergone selective taphonomic processes that conditioned their recovery and identification in the archaeological record [146].

The evolution of local fishing technology also played a role in species variation through time and among sites. For example, increased remains of pelagic species in sites containing ceramic artefacts of the Taquara-Itararé tradition coincided with the appearance and spread of single-piece baited fishing hooks manufactured from mammal bones from 1200 years ago [40]. Groups producing/using ceramic artefacts attributed to Taquara-Itararé tradition also had the technology to colonise oceanic islands in southern Brazil, such as Arvoredo Island located more than 10 km off the mainland [147, 148]. This evidence supports the emerging consensus that some coastal populations intensified fishing in the Late Holocene [40, 127], and pursued offshore resources. In addition to fishing technology, the evolution of coastal ecosystems in southern Brazil over the last 5000 years (a period covering 98% of the analysed data) potentially affected species distribution and local relative abundances, and hence subsistence models through time. As recently discussed by Toso et al. [40], the decrease in relative sea level and the silting of some estuarine-lagoonal water bodies in southern Brazil in the Late Holocene may have disrupted access to key lagoonal resources in the Atlantic Forest coast, forcing some human populations (e.g. Taquara-Itararé groups) to intensify the capture of open sea and pelagic species in more recent times.

### Fishing up marine food web in pre-European times?

Top marine predators (sharks and rays) were particularly targeted in southern Brazil from *ca*. 2200 years ago. Our null model approach based on the frequency of species in Babitonga Bay also revealed that pre-European fisheries predominantly captured species of relatively higher trophic position, in addition to larger body size and body mass, compared to modern fish catches and to local fish assemblages. The results thus suggest that past coastal environments supported more complex food webs than currently exist in the region. High trophic level and large-bodied species were possibly more abundant in the past, allowing for their periodic exploitation by Indigenous populations with relatively simple fishing technology for thousands of years [40].

Compared to pre-European assemblages, modern fish catches in Babitonga Bay have a smaller contribution of key functional groups such as top predators (large-bodied piscivores and macrocarnivores) and a higher abundance of lower trophic level and small-bodied species. Notably, it is only in modern catches that we see planktivores being exploited, as well as the increasing contribution of small-bodied species to catch composition. Although there were no significant differences in trophic levels across periods to suggest the occurrence of fishing down the marine food web (gradual decrease in the mean trophic level of fish in fisheries catches due to preferential removal of top predators, [149]), the identified pattern reveals a significant decrease in the encounter rate with large-bodied and high trophic level species in modern catches. The causes are possibly attributed to overfishing, bycatch and other detrimental impacts on top predators, even though the effects of taphonomic processes and recovery techniques on modelled outputs remain unclear.

### Implications for marine biological conservation

Significantly, Brazil is one of the world’s largest elasmobranch fishing industries, with over 90% of the catches in Brazil obtained by fisheries in Santa Catarina and Rio Grande do Sul [150]. Until the last three decades, southern Brazil’s continental shelf supported a high diversity and large populations of coastal elasmobranchs that were exploited by subsistence, recreational and commercial fisheries [151–153]; however, many of these species are now considered endangered [154]. Other environmental stressors, such as industrial and urban activities [155], including the closure of the Linguado channel in the 1930’s and its impact on migratory species [156, 157], have aggravated local ecological conditions.

Similarly, several species of demersal and pelagic sharks and rays reported in the archaeological record [86, 158–160], have seen reductions in population sizes over the last decades [161, 162], and are currently listed in the Vulnerable (VU), Endangered (EN), Critically Endangered (CR) and/or Regionally Extinct (RE) categories [68]. Long-living species of Sciaenidae and Ariidae are currently considered overexploited with risks of significant catch reduction in the near future, while others have collapsed [26, 163]. A large number of species reported in the archaeological record are currently categorised as Data Deficient (DD) in terms of their distribution and abundance [67, 68] (cartilaginous fish—*Rhinoptera bonasus*, *Carcharhinus altimus*, *Carcharhinus brachyurus*, *Isurus paucus*, *Lamna nasus*; bony fish—*Sardinella brasiliensis*, *Trachinotus cayennensis*, *Anisotremus surinamensis*, *Menticirrhus americanus*, *Menticirrhus littoralis*, and *Pagrus pagrus*). Given their socio-economic importance, notably for the food security and livelihood of local coastal communities (PAN Mangrove [69]), an understanding of species and population responses to long-term fishing pressure is critical for their sustainable management.

Coastal and ocean ecosystems have fuelled subsistence fisheries for thousands of years along the Brazilian coasts. As a result, hundreds of archaeological sites preserve information of past biological diversity of potential interest for fisheries management and conservation debates. Here, we have directed our analyses towards bony and cartilaginous fish, as they represent some of the most commonly occurring and abundant faunal remains in the archaeological sites under examination. Other organisms such as molluscs, echinoderms and crustaceans are also untapped sources of information on long-term human-coastal interaction in this region. Future studies that combine these proxies may enhance our understanding of past Indigenous coastal adaptation.

Our study revealed that demersal species contributed to most of the catches and thus played an important role in past Indigenous food security. Compared to present day fish catches, our study indicates that past encounter rates were possibly greater for species of high trophic level, large body size and body mass to enable their persistent capture and consumption, which invites us to rethink the antiquity of the human footprint on ocean ecosystems in the region. Some of these species are currently threatened by overfishing and habitat degradation, while others are surrounded by uncertainties regarding their modern distribution and abundance. The results presented here provide the most direct evidence of what species have been subjected to long-term fishing efforts, and offer benchmarks of species relative abundances and distributions prior to fish commoditization in the Southwestern Atlantic Ocean.

## Supporting information

S1 Table Archaeofauna references. (XLSX)

S2 Table Archaeological sites data. (XLSX)

S3 Table Ichthyo-archaeofauna qualitative data. (XLSX)

S4 Table Ichthyo-archaeofauna quantitative data. (XLSX)

S5 Table Radiocarbon chronology of archaeological sites. (XLSX)

S6 Table References for modelling fish traits across time periods in Babitonga Bay. (XLSX)

S7 Table Modelled fish traits across time periods in Babitonga Bay. Absence and presence of fish for time periods: 4500–1150 cal BP (Sambaqui), 1050–600 cal BP (Taquara-Itararé), and AD 1994–2015 (modern).
(CSV)

S8 Table Modelled fish traits across time periods in Babitonga Bay. Absence and presence of fish for time periods: 4500–1150 cal BP (Sambaqui), 1050–600 cal BP (Taquara-Itararé), and AD 1994–2015 (modern). Data from Projeto de Monitoramento da Atividade Pesqueira em Santa Catarina (PMAP/SC), http://pmap-sc.acad.univali.br/.
(CSV)

S1 Text R script for modelling fish traits across time periods in Babitonga Bay. (R)
